# Race-Level Reporting of Incidents during Two Seasons (2015/16 to 2016/17) of Thoroughbred Flat Racing in New Zealand

**DOI:** 10.3390/ani12081033

**Published:** 2022-04-15

**Authors:** Michaela J. Gibson, Charlotte F. Bolwell, Erica K. Gee, Kylie A. Legg, Chris W. Rogers

**Affiliations:** 1 School of Veterinary Sciences, Massey University, Private Bag 11-222, Palmerston North 4442, New Zealand; m.gibson@massey.ac.nz (M.J.G.); c.bolwell@massey.ac.nz (C.F.B.); e.k.gee@massey.ac.nz (E.K.G.); 2 School of Agriculture and Environment, Massey University, Private Bag 11-222, Palmerston North 4442, New Zealand; k.legg@massey.ac.nz

**Keywords:** thoroughbred racing, incident, non-incident, steward, stipendiary report, injury, social license

## Abstract

**Simple Summary:**

Retrospective stipendiary stewards’ reports of race day events during the 2015/2016 and 2016/2017 Thoroughbred flat racing season in New Zealand were examined to describe the reasons and outcomes for race day veterinary examinations of horses. Most reports were recorded as non-incident as a result of routine screening or poor performance. The large proportion of poor performance examinations reflected the role of stewards in maintaining racing integrity and animal welfare. The lower incidence of fatalities and injuries reported compared to overseas racing indicates a lower risk profile for New Zealand Thoroughbred racing.

**Abstract:**

The objective of this study was to describe the incident and non-incident reports of Thoroughbred flat racing in New Zealand. Retrospective stipendiary stewards’ reports of race day events during the 2015/2016 and 2016/2017 racing season were examined. The primary injury and reporting outcomes were analysed to assess the horse- and race-level risk factors associated with the occurrence of incident and non-incident reports. The number of incident and non-incident events and binomial exact 95% confidence intervals were calculated per 1000 horse starts. Most reports were for non-incidents and examinations were requested for poor performance (10.3 per 1000 races, 95% CI = 9.5–11.1). Horses running in open-class races had greater odds of having an incident than horses in lower-rating classes. The incidence of musculoskeletal injuries (1.3 per 1000 races, 95% CI = 1.13–1.40) and fractures (0.6 per 1000 races, 95% CI = 0.39–0.74) were low and similar to previous New Zealand reports. There was a low incidence of epistaxis (0.8 per 1000 races, 95% CI = 0.69–0.92) possibly due to trainers screening susceptible horses before entering them in races, due to the regulatory consequences of an episode of epistaxis during a race.

## 1. Introduction

Despite international regulation there is inherent variation between different jurisdictions and countries in the pattern and structure of Thoroughbred racing. These subtle differences appear to influence the both the type and frequency of injury observed. Meta-analysis of catastrophic musculoskeletal injury (CMI) in Thoroughbred racing has highlighted the variation in incidence and risk factors between jurisdictions [1] and the need for jurisdiction-centric research to identify type and frequency of injury observed and the respective risk factors for these.

The structure and pattern of Thoroughbred racing and training in New Zealand has been previously described [2,3] and this work has highlighted a subtle increase in the age and career length of the New Zealand racehorse. In theory these changes should be associated with a shift in the pattern of type and frequency of injuries observed. Earlier studies utilised a selected group of horses that failed to finish a race as a measure of racing integrity and to quantify the epidemiology of musculoskeletal injury (MSI) [4]. However, this dataset was restricted to horses that failed to finish a race and thus represented a subset of horses racing and may have under reported some of the minor veterinary events which require quantification. The subsequent changes in the age profile of the New Zealand racing population may also have resulted in a shift in the reported incidence rates, particularly for musculoskeletal injury.

In New Zealand, the Racing Integrity Board is responsible for the regulatory process across the three racings codes. This regulatory structure provides separation of the running of the racing programme from the regulation of racing and provides a consistent pattern and process of regulation across racing codes and race meetings. To ensure a high level of welfare integrity, many racing jurisdictions use some form of race day examination and supervision in conjunction with race day veterinarians. In Australasia, this regulatory and supervisory role is the task of the stipendiary stewards [5]. At the completion of the race meeting, a summary of the stewards’ activities and the horses of interest examined is generated and published to maintain transparency of racing integrity [4]. Reports require an assessment from the designated veterinarian on duty for the meeting and can be categorised as either non-incident reports or incident reports. A non-incident report is generally requested as part of routine screening of horses, and stewards identify a horse within the race for an examination. In some cases, this may be a horse that did not race up to expectation, based on previous form or relative ranking within the betting. The veterinary examination typically focuses on possible reasons a horse may not have performed up to expectations, or if a horse’s health is questioned. An incident report is the result of an “event” before or during a race (e.g., horse collision, trip or fall) that requires a horse to be examined.

When stipendiary stewards’ reports are combined with race data, the information provides the ability to describe the incidence of injuries (from minor lacerations to catastrophic injury) and calculate the odds of these events or outcomes occurring with different horse and environment level variables [4,6]. Potential risk factors reported for MSI and veterinary events have included track condition, race distance, race class, age of the horse, training intensity and the number of starters [6,7]. By quantifying and describing common injuries and causes of injuries in Thoroughbred racing, targeted research can be undertaken to mitigate the occurrence of incidents. The collection of these data enables the monitoring of industry practice and optimization of horse welfare. These data enable the industry to meet its duty of care to racehorse welfare by making evidence-based changes to the management and structure of racing. By minimising horse injury and loss, the racing industry can meet its social license to operate [8].

To understand racing integrity, both incident and non-incident data needs to be considered. Therefore, the objective of this study was to describe the incident and non-incident reporting by the stipendiary stewards during the 2015/2016 and 2016/2017 New Zealand Thoroughbred racing seasons, the primary injury and reporting outcomes and examine horse- and race-level variables associated with the incidence of these outcomes.

## 2. Materials and Methods

### 2.1. Data Acquisition

The data were obtained for all race starts during the 2015/2016 and 2016/2017 racing season as a Microsoft Excel (Microsoft Corporation, Redmond, WA, USA) spreadsheet from New Zealand Thoroughbred Racing, the official registration body for Thoroughbred racing in New Zealand. An Excel spreadsheet of all stipendiary steward reports for the same Thoroughbred racing seasons was obtained from the Racing Integrity Unit (now renamed as the Racing Integrity Board), the official racing compliance organization for all three racing codes (Thoroughbred, Harness and Greyhound racing) in New Zealand. Each stipendiary stewards’ report recorded the date, racecourse, race number, horse name and other information relating to the non-incident/incident such as the reason for the requested report and the finding of the of the veterinary examination.

Stipendiary stewards’ reports were categorised as either incident reports where screening was due to major events occurring before (from the start of the race meeting), during and after the race, or non-incident reports where screening was due to poor performance or other non-event concerns, often as a part of routine racing integrity screening.

### 2.2. Statistical Analysis

#### 2.2.1. Data Cleaning

To obtain information about racing track condition (fast, good, slow, heavy and dead), race type, horse age and other relevant track and horse information, stipendiary stewards’ reports were cross-referenced with the official race start records and results from the same period by exporting to SAS (version 9.4, SAS Institute Inc., Cary, NC, USA) where the datasets were merged by the horses official race name and date of the race. Apparent errors such as misspelt horse names and incorrect dates were checked manually against the official formal transcript of the relevant stipendiary steward’s report, hosted and archived on the New Zealand Thoroughbred Racing website. Horses that did not race or were miscategorised (harness racing) were removed from the dataset. Five stipendiary stewards’ report records were removed due to misentered values (during computer entry) that prevented linkage between the two databases. Horses that were scratched from the race (n = 139 or 12% of stipendiary stewards’ reports, removed prior to the race starting) were also removed from the dataset. Using the official descriptors provided within the datasheets, reports were coded as incident and non-incident reports.

#### 2.2.2. Data Analysis

Statistical analysis was conducted using SAS version 9.4 (SAS Institute Inc., Cary, NC, USA) for the following analyses: Median values and interquartile range (IQR) were calculated for race distance, number of participants per race, number of starts per horse and number of starts per age category (2, 3, 4+ years old). Chi-Squared tests were used to determine the dependence of the proportion of poor performers per age category with the distribution of age within the population. The odds ratios of incident reporting and 95% confidence intervals at a univariable level were calculated for the number of participants (races with less than or more than nine participants).

The odds ratios of incident reporting and 95% confidence intervals (95% CI) at a univariable and multivariable level were calculated for the variables of race distance (categorised as under 1200 m, 1201–1400 m, 1401–1600 m and 1600+ m), age category, race class (maiden, rating 60–65, rating 70–95 and open), sex (male (colt, stallion or gelding) and female (mare or filly)) and track condition (fast, good, slow, dead and heavy, based on penetrometer readings [9]) using a logistic model. Variables were screened in univariable models and those with *p* < 0.2 were included in multivariable models. Final models were determined based on likelihood ratio test *p* values of *p* ≤ 0.05. The interaction between variables was tested but was not significant (*p* > 0.05), so was removed.

Differences in median values from non-parametric data, such as number of races a horse participated in per season by age category, were tested using a Kruskall–Wallis test.

## 3. Results

### 3.1. Thoroughbred Racing Data

A total of 2683 races were run in the 2015/2016 season and 2460 races in the 2016/2017 season. During the 2015/2016-2016/2017 seasons, 6953 horses had a race start, with 5120 horses participating in at least one race in the 2015/2016 season and 4815 horses participating in at least one race in the 2016/2017 season. Just under half (49.0%) of the racing population were mares and fillies, followed by geldings (47.6%) then stallions and colts (3.3%) (Table 1).

There were a total of 54,690 racing starts, of which 28,708 (52.4%) were during the 2015/2016 season and 25,982 (47.6%) were in the 2016/2017 season. Races were run at 49 racetracks with 37.4% (1923/5143) of races being held in the northern region, 35.5% (1826/5143) of races being held in the central region and 27.1% (1394/5143) being held in the southern region.

Of the 5143 races, the largest proportion of races were run on tracks with the going classified as “dead” (penetrometer reading 2.6–3.5) (1847 races or 35.9%), the remaining races were run on “good” (penetrometer reading 2.0–2.5) (1211 races or 23.5%), “heavy” (penetrometer reading 4.6+) (1068 races or 20.8%), “slow” (penetrometer reading 3.6–4.5) (1009 starts or 19.6%) and “fast” (penetrometer reading 0.5–1.9) (8 races or 0.2%) tracks.

There was a median race distance of 1400 m [IQR 1200–1600] with 27.1% (1394/5143) of races being over 1600 m (1 mile).

There was a median of 11 participants per race [IQR 9–13]. Horses participated in a median of five [IQR 2–8] races per season. Younger horses participated in fewer starts per season, 2-year-olds with a median of two [IQR 1–3] starts per year, 3-year-old horses with a median of four [IQR 2–6] and 4+yo with a median of six [3,4,5,6,7,8,9] starts per year (*p* < 0.001).

### 3.2. Stewards Reports

There were a total of 1020 steward’s reports for horses that participated in a race, of which 179 were coded as incidents and 841 were coded as non-incidents. Three horses had two reports on the same day. Most of the stipendiary steward’s reports described events relating to races (91.1%, 930/1020) rather than those occurring before (6.4%, 65/1020) and after (2.5%, 24.5/1020) the entered race.

Within both the incident and non-incident reports, 31 outcomes coded as musculoskeletal fractures (0.6 per 1000 races, 95% CI = 0.39–0.74), four cardiac failures (0.07 per 1000 races, 95% CI = −0.12–0.3) and 44 bleeders (epistaxis) (1.01 per 1000 races, 95% CI = 0.99–1.03) were recorded.

#### 3.2.1. Incident Reports

There were 3.3 incident reports per 1000 starts (95% CI = 2.87–3.7). Track conditions were reported as dead for 48.0% of incident reports followed by good (21.8%), heavy (17.9%), slow (11.7%) and fast (0.6%).

At the univariable level, there was an association of track condition with the frequency of incident reports (*p* = 0.004) such that an incident was less likely to occur in a race run on a good, slow or heavy track than a dead track (Table 2). There was an association of field size with incident reports such that races with nine or more participants were 1.9 (95% CI = 1.2–3.0) times more likely to have at least one incident (*p* < 0.01). Race distance and age category had no effect on the odds of having an incident (Table 2). Horses in rating class races had lower odds of an incident occurring than horses in open (higher grade) races. Female horses had higher odds of an incident compared to males (Table 2).

In the final multivariable model, race class, sex and track condition were significantly associated with the odds of an incident occurring during a race (Table 3).

There were a variety of reasons listed for requesting an incident report with the main descriptor being horses that were pulled up (0.27 per 1000 starts (95% CI = 0.05–0.50); 8.4%), followed closely by horses that fell (0.24 per 1000 starts [0.01–0.47]; 7.3%). However, most incident reports did not have a descriptor of the event or were reported as “other” (64.8%). The most common clinical outcome from an incident report was no observable abnormalities detected (NOAD) (38.55%), followed by laceration/abrasion (21.23%). Musculoskeletal fractures were reported in 5.59% (n = 10) of incident reports and cardiac failure was reported in 1.68% of incident reports. The reporting rates of clinical observations across all incident stipendiary reports are presented in Table 4.

#### 3.2.2. Non-Incidents Reports

A non-incident report occurred 15.4 times per 1000 starts [IQR 14.4–16.4]. The major reason (67.2%, n = 566/841) for the request of a non-incident report was for the routine post-racing screening of horses, or for health concerns not related to an “event” (10.3 per 1000 races, 95% CI = 9.5–11.1); for example, the screening of horses who performed below expectations. Stewards were responsible for requesting most poor performance reports (85.3%, n = 483/566). Stewards were 2.7 [95% CI = 1.9–3.8] times more likely to request a non-incident report for poor performance than for any other reason (*p* < 0.001). The distribution of age across all poor performers reflected the underlying age distribution (*p* = 0.005). Over the two seasons, 70.5% (n = 399/566) of poor performers were aged between 3 and 6 years of age. There was a maximum of three reports for poor performance per horse per season with fifteen horses having two reports and three horses having three reports for poor performance in one season. There was a positive association of field size with non-incident reports such that a non-incident report was 1.4 [95% CI = 1.2–1.8] times more likely to be requested in races with nine or more participants.

The largest category for clinical outcome of a poor performance exam was NOAD during screening (59.4%, n = 336/566). These were limited clinical outcomes classified as poor recovery (6.0%), arrhythmia/cardiovascular (8.0%), respiratory issues (5.5%), musculoskeletal injuries (5.3%) and lacerations/abrasions (3.7%).

The largest category of findings from non-incident reports was NOAD (46.7%). The main clinical findings were laceration/abrasion (46.7%) followed by lameness (7.5%) and MSI issues that were not fractures (6.3%). Musculoskeletal fractures were reported in 2.5% (n = 21) of all non-incident reports.

The reporting rates of clinical observations across all non-incident stipendiary reports are presented in Table 4.

## 4. Discussion

The consistency of the regulatory process was reflected in the same frequency of the non-incident reporting between the Thoroughbred and harness racing codes and the increase in odds of a non-incident screening report with increasing field size. Trainers and race officials in the Thoroughbred code were more likely to request a non-incident report than in Harness racing, where the majority of requests for non-incident reports were initiated by the stewards (95%) [10].

No observable abnormalities detected (NOAD) was the most common reporting across both codes and reflects the role of the non-incident reporting as a regulatory screening process. Lacerations and abrasions were the next most frequently reported finding, followed by lameness. Relatively few horses were reported as bleeders (epistaxis) with the prevalence being approximately half the value reported in Australia on a per start value [5], or per horse [11], or from the United Kingdom [7]. This lower value does not reflect the increasing age profile of the New Zealand Thoroughbred racing population [12], which theoretically should be associated with a greater incidence of epistaxis than reported here. Rather, the low level of epistaxis reported may reflect caution by New Zealand trainers in presenting a horse for racing that they suspect may have an episode of epistaxis. Under the New Zealand rules of racing (Article 651) if a horse suffers an initial episode of epistaxis at a race meeting it must not be ridden for two months, may not start in a trial or race start for three months and may only race after being cleared by a stipendiary steward and/or a veterinarian. A subsequent episode would result in the horse being banned from racing in New Zealand.

The higher rate of incident reports in Thoroughbred racing compared to harness/Standardbred racing [10], may reflect differences between the racing codes in the speed and pattern of racing and therefore the associated risk of an incident or event during a race and the type of injury. Thoroughbreds had a higher rate of all the musculoskeletal injury categories coded (lame, MSI fracture, other MSI issues) at approximately twice the rate reported in harness racing. However, in comparison to reporting from other racing jurisdictions these rates appear low and align with those reported out of Australia, which reflects the similarity in the pattern of training, racing and regulation between Australia and New Zealand [1,6].

The MSI fracture rate based on the stewards reports was only moderately greater than that previously reported in an earlier study using a more restrictive dataset of horses that failed to finish a race (0.6/1000 vs. 0.48/1000 starters, respectively) [6]. The consistency of this rate is interesting as there has been a subtle increase in the age of the New Zealand racing population with longer racing careers (one extra preparation), which was predominantly driven by geldings staying in work. Increasing age and males (geldings) are reported as increasing the odds of fracture and CMI across many studies [1]. However, in some studies the relationship of age with fracture and CMI were quadratic rather than linear, indicating a “healthy horse” effect within the population [1].

The differences in fracture rate between Thoroughbred racing and harness racing may reflect the pattern of training and racing and the respective magnitude of load during each limb load cycle. Thoroughbred horses participated in fewer starts per season than harness racing horses (five vs. seven starts), which theoretically should result in a lower number of loading cycles during racing and training. However, the number of load cycles is only part of the risk profile and the magnitude and type of loading in the limb from galloping rather than trotting/pacing, which applies greater stresses, would contribute to a greater risk of fracture as fatigue life decreases exponentially with increasing stress [13].

In Thoroughbreds the majority of fractures tend to be in the distal limb in bones such as the third metacarpal and third metatarsal [7]. On grass tracks the predominant fracture presentation in these bones are condylar fractures, many of which can be non-displaced, which may explain the incidence of musculoskeletal fractures in the non-incident reports and not associated with a perceived “event”. Often these fractures are found when the horse is examined for poor performance, or the horse becomes lame after the race has concluded. Fractures of this nature tend to be less noticeable until closer inspection.

A horse had lower odds of an incident racing on a slow track compared to other track conditions. A similar pattern of lower risk for slow and good tracks has been reported for fracture and CMI [1]. In New Zealand there are few occasions when tracks are presented as fast, generally representing less than 1% of all races [14]. In the current study, fast track data only contributed 0.2% of the race data and provided 0.6% of the incident data. This low frequency of racing on fast tracks prevented identification of the risk associated with fast tracks. It may be that the reduction in the number of races presented as fast tracks is a direct response by the industry to the perceived risk of racing on such surfaces [15,16].

The higher odds of incidents in open-class races may reflect the intensity of racing in the higher-grade races and the greater regulatory scrutiny of events associated with races with significant stakes prize money. Open races are restricted to more experienced horses, but are more competitive, as horses are run at a faster pace, potentially contributing to a higher risk of an incident than lower-rating classes. Maiden and lower-grade races have a greater proportion of younger or inexperienced horses. However, within New Zealand it is the cohort of experienced jockeys that ride the younger horses and make up the majority of rides in the other grades of racing [17]. This continuity of most races consisting of experienced jockeys, and the use of trials (non-totaliser/qualifying races) by trainers to provide additional experience prior to race day [4,12], may provide an explanation for the lack of differentiation of risk in the other race grades. Data reporting incidence of MSI injuries in Australia, which has a similar pattern and structure of racing to New Zealand, identified a greater incidence of MSI injuries on metropolitan tracks compared to country tracks and in stakes races compared to non-stakes races [18]. Metropolitan tracks hold more prestigious races (greater stakes and black type races) than country racetracks, attracting horses and jockeys of a higher calibre [19]. A similar trend is also reported in Harness racing data from Canada, where higher-ranking tracks were associated with a higher incidence of sudden death and horses failing to finish compared to lower-ranking tracks [20].

The data in this study originated from the completion of *pro forma* forms, which provided prompts and permitted some variation in the level of detail reported. This form provided data with a variety of descriptors for many conditions and limited detailed quantification of the site or the underlying pathology that resulted in the lameness recorded. Further confounding the description and identification of lameness immediately after the race is the fact that many injuries or pathologies associated with lameness are often not easily observable until the horse has cooled down, often some hours post-race, or even on the day after the race [21]. Under current practise and regulations for racing in New Zealand the trainer is not obliged to notify racing authorities of lameness outside the regulatory timeframe of the race day.

During the 2018/19 season an online structured reporting system was introduced for the race day stewards. This system was a copy of the Australian Racing injury database system and reflects the greater integration of regulatory processes and reporting between New Zealand and Australia. Data derived using the online system should provide greater consistency in descriptors and reporting, and the ability to compare data between New Zealand and the Australian jurisdictions.

The primary issues associated with the social license to operate in all horse racing disciplines tends to focus on the concept of injury and risk of injury to the equine participants [8]. Routine screening data (i.e., stewards’ reports) provide metrics for industry performance. The level of steward’s reporting in Thoroughbred flat racing in New Zealand indicates that these data are representative of the industry and provides robust metrics of the industry’s performance. The low incidence of significant clinical findings from this high level of screening and reporting indicates that Thoroughbred flat racing in New Zealand is meeting its duty of care to the horses racing in it and the primary issues associated with the social license to operate within horse racing.

## 5. Conclusions

The high level of reporting within the Thoroughbred racing industry during the 2015/2016 and 2016/2017 seasons reflects the need for industry transparency. Incidents reported by stewards were more likely to occur in open grade races, reflecting the intensity of racing in these grades. The incidence of fracture was similar to previous reports and confirms the lower than expected rate within New Zealand racing compared to overseas studies, possibly due to few races held on tracks with surface conditions rated as fast. The lower than expected incidence of epistaxis may be due to the reluctance of trainers to enter horses in a race if they suspect it may be susceptible to epistaxis, due to the strong regulatory control of this condition. The prominent reporting by stewards reflects the important part stewards play in maintaining racing integrity and meeting the social license to operate.

## Figures and Tables

**Table 1:** Number of horses participating in the 2015/2016 and 2016/2017 New Zealand Thoroughbred racing season, by age and sex.

	Horses per Season and Starters			Sex				
Age (Years)	2015/16	2016/17	Starts	Colts	Fillies	Geldings	Entires/ Stallions	Mares
2	362	339	1603	127	405	184		
3	1401	1213	11,693	130	1450	1073		
4	1347	1359	16,098			1255	46	1420
5	870	868	11,651			896	18	838
6	592	491	7208			596	7	492
7	327	303	4099			443	2	207
8	126	148	1527			231		60
9	64	55	529			120		11
10	20	26	162			58		1
11	8	7	83			24		
12	2	5	34			12		1
13	1	1	3			7		
Total	5120	4815	54,690	257	1855	4899	73	3030

**Table 2:** Univariable odds ratios and 95% confidence of an incident occurring with the effects of age, distance, race class, sex and track condition.

	Odds Ratio [95% CI]	Wald *p*-Value	*p*-Value
Age category (years)			
2	Reference	0.401	
3	1.0 [0.4–2.4]		0.949
4+	0.8 [0.4–1.9]		0.634
Distance			
Under 1200 m	Reference	0.459	
1201–1400 m	1.0 [0.7–1.5]		0.967
1401–1600 m	1.0 [0.7–1.5]		0.880
1601+ m	−0.3 [0.2–1.7]		0.186
Race class			
Open	(Reference)	0.034	
Maiden	0.7 [0.5–1.0]		0.071
Rating 60–65	0.6 [0.4–0.9]		0.012
Rating 70–95	0.5 [0.3–0.9]		0.029
Sex			
Male (colt, gelding or stallion)	(Reference)	0.022	
Female (filly or mare)	1.4 [1.1–1.9]		0.022
Track condition			
Dead	(Reference)	0.004	
Fast	3.8 [0.5–27.8]		0.187
Good	0.7 [0.5–1.0]		0.052
Slow	0.4 [0.3–0.7]		0.001
Heavy	0.7 [0.5–1.0]		0.038

**Table 3:** Multivariable odds ratios and 95% confidence of an incident occurring with the effects of race class, sex and track condition.

	Odds Ratio [95% CI]	Wald *p*-Value	*p*-Value
Race class, Sex and Track condition		0.001	
Race class			
Open	(Reference)	0.029	
Maiden	0.7 [0.5–1.0]		0.048
Rating 60–65	0.6 [0.4–0.9]		0.011
Rating 70–95	0.5 [0.3–0.9]		0.030
Sex			
Male (colt, gelding or stallion)	(Reference)	0.030	
Female (filly or mare)	1.4 [1.0–1.9]		0.030
Track condition			
Dead	(Reference)	0.011	
Fast	4.0 [0.5–29.2]		0.172
Good	0.7 [0.5–1.0]		0.037
Slow	0.5 [0.3–0.8]		0.004
Heavy	0.7 [0.5–1.0]		0.124

**Table 4:** Frequency of clinical findings in incident (n = 179) and non-incident (n = 841) stipendiary reports over two complete Thoroughbred racing seasons. Data are reported as frequency per 1000 starts (with 95% confidence intervals) and the percentage of clinical findings within the respective report type (incident or non-incident).

	Incident Reports		Non-Incident Reports	
Description	Frequency per 1000 Starts	%	Frequency per 1000 Starts	%
Arrhythmia	0.16 [−0.08–0.41]	5.03	0.75 [0.62–0.88]	4.88
Cardiac failure	0.05 [−0.20–0.31]	1.68	0.02 [−0.24–0.28]	0.12
Laceration/abrasion	0.69 [0.55–0.84]	21.23	2.23 [1.94–2.52]	14.51
Lame	0.18 [−0.06–0.42]	5.59	1.15 [1.05–1.26]	7.49
MSI fracture	0.18 [−0.06–0.42]	5.59	0.38 [0.18–0.59]	2.50
Other MSI issues	0.29 [0.07–0.52]	8.94	0.97 [0.92–1.02]	6.30
No observable abnormalities detected	1.26 [1.13–1.40]	38.55	7.19 [6.53–7.85]	46.73
Poor recovery	0.05 [−0.20–0.31]	1.68	0.79 [0.66–0.91]	5.11
Respiratory issues	0.07 [−0.18–0.33]	2.23	0.62 [0.46–0.78]	4.04
Previous injury	0.04 [−0.22–0.30]	1.12	0.05 [−0.20–0.31]	0.36
Bleeders (epistaxis)	0.20 [−0.04–0.44]	6.15	0.80 [0.69–0.92]	5.23
Miscellaneous	0.07 [−0.18–0.33]	2.23	0.42 [0.22–0.62]	2.73

## Data Availability

Source data is publicly available via respective websites, collated extracts are the property of respective bodies.
